# BreCAN-DB: a repository cum browser of personalized DNA breakpoint profiles of cancer genomes

**DOI:** 10.1093/nar/gkv1264

**Published:** 2015-11-19

**Authors:** Pankaj Narang, Parashar Dhapola, Shantanu Chowdhury

**Affiliations:** 1 School of Computational and Integrative Sciences, Jawaharlal Nehru University, New Delhi, India; 2 GNR Center for Genome Informatics, Institute of Genomics and Integrative Biology, CSIR, Delhi, India; 3 Proteomics and Structural Biology Unit, Institute of Genomics and Integrative Biology, CSIR, Delhi, India

## Abstract

BreCAN-DB (http://brecandb.igib.res.in) is a repository cum browser of whole genome somatic DNA breakpoint profiles of cancer genomes, mapped at single nucleotide resolution using deep sequencing data. These breakpoints are associated with deletions, insertions, inversions, tandem duplications, translocations and a combination of these structural genomic alterations. The current release of BreCAN-DB features breakpoint profiles from 99 cancer-normal pairs, comprising five cancer types. We identified DNA breakpoints across genomes using high-coverage next-generation sequencing data obtained from TCGA and dbGaP. Further, in these cancer genomes, we methodically identified breakpoint hotspots which were significantly enriched with somatic structural alterations. To visualize the breakpoint profiles, a next-generation genome browser was integrated with BreCAN-DB. Moreover, we also included previously reported breakpoint profiles from 138 cancer-normal pairs, spanning 10 cancer types into the browser. Additionally, BreCAN-DB allows one to identify breakpoint hotspots in user uploaded data set. We have also included a functionality to query overlap of any breakpoint profile with regions of user's interest. Users can download breakpoint profiles from the database or may submit their data to be integrated in BreCAN-DB. We believe that BreCAN-DB will be useful resource for genomics scientific community and is a step towards personalized cancer genomics.

## INTRODUCTION

It is widely understood that incidence of structural genomic alterations (GAs) is frequently associated with cancer and indeed has been posed as a prominent cause for acquisition of hallmarks of cancer initiation/progression (1,2). These GAs could be large-sized deletions, insertions, inversions, translocations, fusions and copy number variations (3–5). Studies indicate that GAs not only account for larger genomic heterogeneity in populations but may also have a much larger than anticipated impact on oncogenesis and cancer progression (6,7). Most studies so far have focused mainly upon association of mutations, especially non-synonymous point mutations present in the coding regions of the genome. Results from such studies have shown that though certain point mutation events in cancer are more associated with specific genes like *BRCA1* in breast cancer (8), *TP53* in glioblastoma multiforme (9), such mutations are present in only a small percentage of disease cases (10). This has led to an increased interest in large-sized GAs (that may span from tens of bases to few megabases of genome) to better understand cancer etiology.

Profiling GAs have been more challenging than profiling point mutations, mainly due to requirement of deep coverage genome sequence data and algorithms that can map sequence reads to identify breakpoints at single base resolution. With fulfilment of these requirements, various efforts to elucidate the role of GAs in cancer genome have been made. However, they were unable to include either large sample size, single base resolution or/and accessible data for individual breakpoint profile. With these features in mind, BreCAN-DB was developed to allow users navigate genomes for mining individualized single base resolution breakpoint events in a readily scalable setting.

Recent studies involving meta-analysis of large-scale data sets have indicated the utility of integrating and methodologically making statistical analysis to gain biological insights; these span from analysis of structural variants at low resolution, gene expression and small RNA expression (11–13). Moreover, in last few years, various prominent resources have been developed such as COSMIC (14), The Cancer Genome Atlas (TCGA) data portal (https://tcga-data.nci.nih.gov/tcga/), UCSC Cancer Genomics Browser (15) and International Cancer Genome consortium (ICGC) data portal (16), serving data related to genomic variations in cancer both as pooled datasets as well as at personalized level. Of these, COSMIC uses a gene-focused approach where most mutation data are available for genes nominated from Cancer Gene Census (17) while TCGA and ICGC provide genomic SNP and copy number profiles at personalized level. BreCAN-DB complements these databases by adopting an individual profile-based approach for large-sized GAs over gene-based approach, so that information on low recurrence but potentially relevant events is retained. It is being increasingly appreciated to understand genome in a personalized context. Also, many of the genomic variations have already been associated with response to therapy while many others still remain elusive (18). Hence, it is crucial that each cancer's structural variation is studied individually so that viable targets for precision therapy may be developed.

To facilitate studies involving DNA breakpoints at personalized levels, we have made BreCAN-DB: ‘**BRE**akpoint profiles of **CAN**cer genomes **D**ata**B**ase’, a browsable repository of personalized cancer DNA breakpoint profiles mapped over the entire genomes of 99 cancer and matched control pairs from five cancer types *viz*. glioblastoma multiforme (GBM), breast invasive carcinoma (BRC), lung adenocarcinoma (LUAD), ovarian serous cystadenocarcinoma (OV) and head and neck squamous cell carcinoma (HN). We also provide functionality of visualizing and comparing DNA breakpoint profiles between samples of same cancer type or across different cancer types using BreCAN-DB.

## MATERIALS AND METHODS

### Implementation

BreCAN-DB has been implemented in three phases: Phase 1- collection of whole genome sequencing data of cancer and matched normal tissues, Phase 2- mapping of somatic DNA breakpoint profiles and breakpoint hotspots and Phase 3- user interface development for interactive visualization through web browser. These three phases are described below.

### Data collection

**Figure 1.**
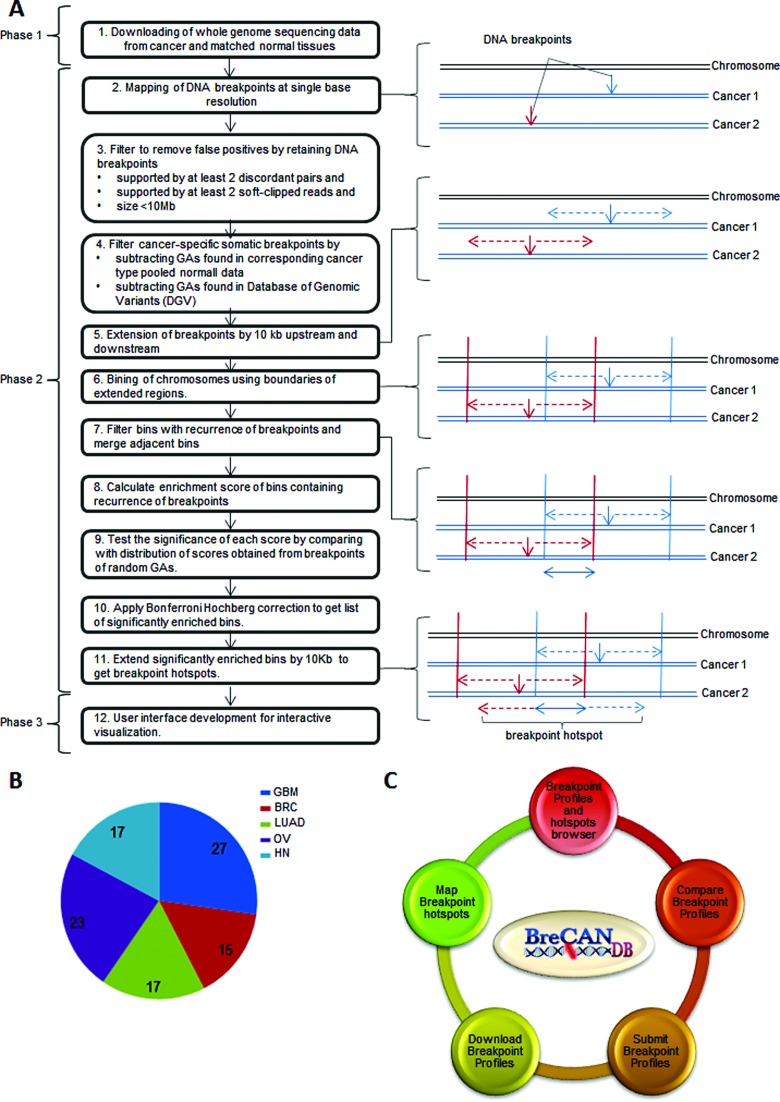
(**A**) Three phases for the development of BreCAN-DB: Phase 1- collection of whole genome sequencing data from cancer and matched normal tissues; Phase 2- mapping of breakpoint profiles and breakpoint hotspots; and Phase 3- designing a web-accessible and interactive user interface. (**B**) Pie chart representing number of cancer-normal pairs in five types of cancer. (**C**) Five modules in BreCAN-DB.

To generate DNA breakpoint profiles, we retrieved the Illumina whole genome paired end sequencing reads from 99 cancer-normal pairs comprising five cancer types *viz*. 15 pairs of BRC from Banerji et. al (19), 27 pairs of GBM (9), 17 pairs of LUAD (20), 23 pairs of OV (21) and 17 pairs of HN (22) from The Cancer Genome Atlas (phase 1 of Figure 1A). Sequencing data for GBM, LUAD, OV and HN were downloaded in the BAM format (23), while data for BRC were downloaded in SRA (sequence read archive) format from the database of Genotypes and Phenotypes (24) (dbGaP Study Accession: phs000369 and phs000178). The paired-end sequencing reads of BRC were aligned to the human reference genome (UCSC build hg19) using BWA aligner with default parameters to create BAM files (25). Further, we also incorporated data of 138 cancer genomes from Yang et al. where breakpoints were called on cancer genomes using Meerkat (26).

### Mapping of breakpoint profiles

Breakpoints of GAs were identified in all normal and cancer genomes independently using Meerkat with default parameters (26). The algorithm of Meerkat calls GAs at positions where cluster of read pairs are discordant and subsequently, the breakpoints of these GAs are precisely determined at single nucleotide resolution using soft-clipped reads. These GAs include insertions, deletions, intra and inter-chromosomal translocations, inversions, tandem duplications and combination of events. To reduce the number of false positives, we filtered GAs, supported by at least two discordant pairs and two soft-clipped reads (phase 2 of Figure 1A). GAs with size more than 10 Mb were also filtered out to get a final list of high confidence GAs. GAs specifically present in only cancer genomes (somatic GAs) were identified by subtracting GAs of all normal genomes from each cancer genome using Meerkat. Further, these GAs were filtered using Database of Genomic Variants (27) to get list of only cancer-specific GAs.

### Mapping of breakpoint hotspots

To identify DNA breakpoint hotspots, we extended breakpoints of each cancer-specific GA by 10 Kb upstream and downstream (phase 2 of Figure 1A). Further, using boundaries of extended breakpoint regions from all cancer genomes, we segmented the reference genome into non-overlapping bins of unequal sizes. The bins that overlapped with extended regions in more than one cancer genome were identified and their enrichment score was calculated as number of samples harbouring extended breakpoint regions overlapping with these bins. Next, we compared these scores with expected distribution of score calculated by randomizing the GAs of all samples 10000 times and calculated their significance. The bins with *P*-value <0.05 after Bonferroni Hochberg correction for multiple hypothesis testing were finally selected as cancer breakpoint hotspots. Similar strategy was used to find breakpoint hotspots for all five types of cancer separately.

### Development of web-accessible interface

The interactive front end interface of database was designed using HTML, CSS and JavaScript and made web-accessible through Apache2 HTTP server running on CentOS7 Linux platform (phase 3 of Figure 1A). The server is hosted at Institute of Genomics and Integrative Biology and can be accessed at http://brecandb.igib.res.in. For visualization of breakpoint profiles in BreCAN-DB, these breakpoint profiles were converted to json format and integrated with JBrowse, an advanced, open source and JavaScript-based genome browser (28,29). JBrowse uses an approach where most feature rendering is done by the client machine rather than server, resulting in quick response to the user. Further, other modules to submit and download breakpoint profiles in BED format were implemented using PHP. The modules ‘Compare Breakpoint profiles’ to compare a new genomic profile against breakpoint profiles of database and ‘Map Breakpoint hotspots’ to map breakpoint enriched regions were implemented using BEDTools (30) and the former outputs sortable barplots, implemented using D3.js (31).

## RESULTS

### Database content and statistics

BreCAN-DB is DataBase of personalized somatic DNA BREakpoint profiles of CANcer genome. The current version of the database comprises whole genome somatic breakpoint profiles of 99 cancer-normal pairs from five types of cancer obtained from The Cancer Genome Atlas and dbGaP (19). The raw breakpoints were mapped at single nucleotide resolution in cancer genome deep sequencing data using Meerkat software (26), which were further filtered using GAs from corresponding cancer type pooled normal data and Database of Genomic variants (27) (see Methods; Figure 1A). These profiles were named as GBM_profile1 to GBM_profile27 for 27 breakpoint profiles of GBM, BRC_profile1 to BRC_profile15 for 15 BRC, LUAD_profile1 to LUAD_profile17 for 17 LUAD, OV_profile1 to OV_profile23 for 23 OV and HN_profile1 to HN_profile15 for 15 HN as shown in Figure 1B. These DNA breakpoints correspond to large-sized deletions, tandem duplications, insertions, inversions, intra and inter-chromosomal translocations and combination of such events. A total of 1 92 626 breakpoints were identified with average number of 1945 breakpoints per cancer genome. Table 1 provides the number of breakpoints in different cancer genomes. Further, we have also mapped significant breakpoint hotspots in five different types of cancer, i.e. genomic regions with more number of observed DNA breakpoints in comparison to expected (see Methods; Figure 1A). BreCAN-DB also collates 4415 breakpoint hotspots distributed over the whole genome (see Table 2). Further, we included 138 whole genome breakpoint profiles from 10 cancer types mapped by Yang et al. (26). This data set included three cancer types already present in our analysis *viz*. breast invasive carcinoma (BRCA; *n* = 35), glioblastoma multiforme (GBM; *n* = 16) and ovarian serous cystadenocarcinoma (OV; *n* = 9); beyond these it included seven other cancer types *viz*. colorectal adenocarcinoma (CRC; *n* = 14), multiple myeloma (MM; *n* = 7), prostrate adenocarcinoma (PR; *n* = 7), hepatocellular carcinoma (HCC; *n* = 19), lung squamous cell carcinoma (LUSC; *n* = 18), uterine corpus endometrioid carcinoma (UCEC; *n* = 10) and kidney renal clear cell carcinoma (KIRC; *n* = 3).

**Table 1.** Summary of data sets used

Cancer type	Source	Number of cancer-normal pairs	Number of Breakpoints	Number of breakpoints per cancer genome
Glioblastoma multiforme (GBM)	TCGA (9)	27 pairs	55 874	2069
Breast invasive carcinoma (BRC)	Banerji et al. (19)	15 pairs	22 077	1471
Lung adenocarcinoma (LUAD)	TCGA (20)	17 pairs	36 086	2122
Ovarian serous cystadenocarcinoma (OV)	TCGA (21)	23 pairs	56 435	2453
Head and neck squamous cell carcinoma (HN)	TCGA (22)	17 pairs	22 154	1303
Total		99 pairs	1 92 626	1945

**Table 2.** Summary of breakpoint hotspots

Cancer type	Number of hotspots	Length of genomic region (Mb)
GBM	839	36
BRC	759	31
LUAD	846	37
OV	990	45
HN	981	43
Total	4415	192

Apart from serving as repository, we have included five modules in BreCAN-DB that help users analyse their own data with breakpoints present in the database. These five modules are ‘Breakpoint Profiles and hotspots browser’, ‘Map Breakpoint hotspots’, ‘Compare Breakpoint Profiles’, ‘Submit Breakpoint Profiles’, ‘Download Breakpoint Profiles’ (Figure 1C), these have been discussed further in the following sections.

### Breakpoint Profiles and hotspots browser

**Figure 2.**
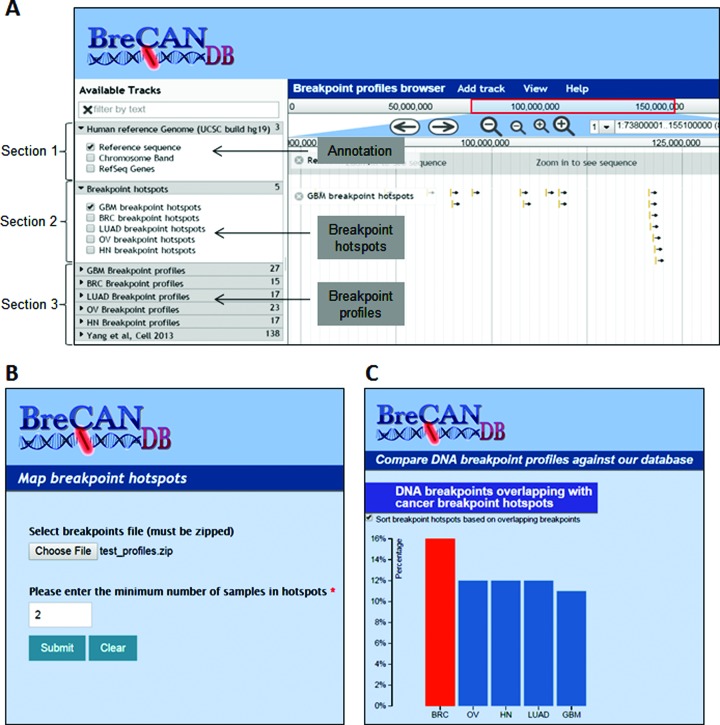
(**A**) A view of ‘Breakpoint Profiles and hotspots Browser’ module in BreCAN-DB representing tracks of overlapping genes, breakpoint profiles and hotspots. The tracks in browser are divided into three sections: Section 1- tracks of human reference genome assembly hg19, chromosome bands and Refseq genes; Section 2- tracks of breakpoint hotspots. Each rectangular bar in these tracks represents one breakpoint hotspot; and Section 3- tracks of individual breakpoint profiles. Each rectangular bar in these tracks represents a 20 Kb region centralized at the breakpoint position. (**B**) ‘Map Breakpoint hotspots’ module takes breakpoint profiles as input and maps regions with breakpoint in at least given number of samples. (**C**) ‘Compare Breakpoint Profiles’ module compares user provided breakpoint profile against breakpoint hotspots and profiles present in the database and shows overlaps in the form of sortable bar plots.

To visualize the distribution of DNA breakpoint profiles of cancer genomes, we provided a genome browser as shown in Figure 2A. The browser tracks have been divided into mainly three sections. Section 1 contains the tracks of human reference genome (assembly hg19), chromosome bands and Refseq genes downloaded from UCSC. Section 2 contains five tracks, each representing breakpoint hotspots (see Methods for details) for one type of cancer. Section 3 contains tracks of personalized whole genome breakpoint profiles, each representing breakpoint profile of a sample from particular cancer type. A separate tab was created in this section to include data from external sources (currently from Yang et al.).

The views are available from chromosome level to nucleotide level. For better visualization, each DNA breakpoint in breakpoint profile tracks has been shown as rectangular bar representing a 20 Kb region centralized at the breakpoint. Moreover, information on chromosome banding is also provided as separate track. Any of these tracks can be switched on or off by user for interactive visualization. The genomic sequence of hotspots and breakpoints can also be obtained by clicking on the respective feature. Also, we have annotated each breakpoint such that its associated genomic alteration type is directly visible to the user. It also allows user to upload breakpoint profiles in Browser Extensible (BED) format (a flexible format that allows features to be represented in form of genomic start and end coordinates) and visualize it along with one or more selected profiles already available in the database. In this way, user can directly compare whether the uploaded breakpoints overlap with his gene of interest or breakpoint hotspots or breakpoint profile of any of the five types of cancer.

### Map breakpoint hotspots

We also provide a module wherein user can submit DNA breakpoint profiles of multiple samples and obtain breakpoint hotspots, regions with breakpoints in more than given number of samples (Figure 2B). The breakpoint profiles in BED format must be zipped with one file per sample and uploaded to the module. These breakpoints should be provided at nucleotide base resolution as this module first extends each breakpoint by 10Kb upstream and downstream. Next, it divides the human genome into non-overlapping bins of unequal sizes using boundaries of extended regions and finds bins that overlap with more than threshold number (given by user) of samples. The output file contains genomic coordinates of breakpoint hotspots along with number of samples having overlap of extended regions.

### Compare breakpoint profiles

One of the important modules of BreCAN-DB allows one to compare user-uploaded new breakpoint profiles against hotspots or individual profiles already present in database (Figure 2C). The breakpoint profiles can either be uploaded as a file from local machine or paste in BED format. User can select type of cancer against which given breakpoint profile will be compared. On submitting data, module reports number of overlapping breakpoints with the hotspots and with the breakpoint profiles as sortable bar plots.

### Submit breakpoint profiles

For further populating the database, we have included a module wherein user can submit whole genome breakpoint profiles of different cancer genomes which will be made open for scientific community after curation. While submitting such profiles, we request submitters to describe methods to map breakpoints clearly in order to reduce method dependent biasing of the results.

### Download breakpoint profiles

We also provide a ‘Download’ module to download all the 99 whole genome breakpoint profiles in BED format. This will be useful for users who want to locally run analysis through integrating BreCAN-DB data sets with their own data.

## CONCLUSION AND FUTURE DEVELOPMENTS

Each cancer genome has unique set of structural GAs, SNPs and other genomic variations and keeping this in mind, BreCAN-DB has been developed as a unique database that not only allows user to visualize individual DNA breakpoint profiles across cancer types but also perform comparative study of their samples against existing data. Through BreCAN-DB, we provide a platform that is readily scalable for further sample's data and allows study of somatic DNA personalized breakpoint at single nucleotide resolution. In summary, we have first developed a curated data set representing high-resolution breakpoint profiles for 99 cancer-normal pairs. Secondly, we systemically identified hotspot regions across cancer types using a novel strategy. Thirdly, we made these data accessible through an integrated next-generation genome browser. Fourthly, we created a platform, where users can perform comparative cancer genomics by checking intersection between their and BreCAN-DB data sets. These data sets can easily be extended by addition of more breakpoint profiles requiring no additional analyses or change in existing data sets as demonstrated through inclusion of data set from Yang et al. (26).

In our data set, we noted that the mean number of breakpoints were higher than previously reported (26). Since we used the same software as used in previous study (Meerkat) for profiling breakpoints, it is unlikely that this difference is due to methodology of mining breakpoints. Rather, we speculate that this might be a sample specific effect as the number of breakpoints was variable across samples in both studies. Furthermore, we found that the average number of genomic alterations in our study (excluding translocations and inversions) were similar to the ones noted earlier (this study did not report translocations and inversions) (32).

We request users to help scientific community by submitting such breakpoint profiles from their study to BreCAN-DB. We believe that this database will prove useful for understanding large-scale personal cancer genomics data.

## AVAILABILITY

The database is freely available at http://brecandb.igib.res.in.
